# Precision Population Cancer Medicine in Brain Tumors: A Potential Roadmap to Improve Outcomes and Strategize the Steps to Bring Interdisciplinary Interventions

**DOI:** 10.7759/cureus.71305

**Published:** 2024-10-12

**Authors:** Umesh Velu, Anshul Singh, Roselin Nittala, Johnny Yang, Srinivasan Vijayakumar, Chanukya Cherukuri, Gregory R Vance, John D Salvemini, Bradley F Hathaway, Camille Grady, Jeffrey A Roux, Shirley Lewis

**Affiliations:** 1 Department of Radiotherapy and Oncology, Kasturba Medical College, Manipal, Manipal Academy of Higher Education, Manipal, IND; 2 Radiation Oncology, University of Mississippi Medical Center, Jackson, USA; 3 Cancer Care, Cancer Care Advisors and Consultants LLC, Ridgeland, USA

**Keywords:** big data, brain tumors, cancer prevention, genomic medicine, glioblastoma multiforme, precision medicine, precision population medicine

## Abstract

Brain tumors, a significant health burden, rank as the second leading cause of cancer among adolescents and young adults and the eighth most common cancer in older adults. Despite treatment advances, outcomes for many brain tumor types, especially glioblastoma multiforme (GBM), remain poor. Precision population cancer medicine (PPCM) offers promising avenues for improving outcomes in brain tumor management. This comprehensive review delves into the current landscape of brain tumor diagnosis and treatment, with a primary focus on the potential of PPCM to enhance care. The review explores several key areas where PPCM approaches show promise. In genetics and molecular biology, the genetic heterogeneity of brain tumors poses challenges and opportunities for targeted therapies. Understanding genetic patterns can guide treatment strategies and improve prognostication. Epigenetic modifications are crucial in brain tumor development and progression. Deoxyribonucleic acid (DNA) methylation patterns, particularly of the O6-methylguanine-DNA methyltransferase (MGMT) gene promoter, serve as essential biomarkers for treatment response and prognosis in GBM. Targeting epigenetic mechanisms could lead to novel therapeutic approaches. Non-invasive liquid biopsy techniques show potential for diagnosis, monitoring, and prognostication in brain tumors. Analysis of circulating tumor DNA and microRNAs may provide valuable information about tumor characteristics and treatment response. Advanced imaging techniques, including radiomics and radiogenomics, combined with artificial intelligence (AI) algorithms, are enhancing tumor detection, characterization, and treatment planning. These technologies can contribute to more personalized treatment approaches. In addition, emerging nanotherapeutic platforms, which involve the use of nanoparticles to deliver drugs directly to tumors, and theranostic approaches, which combine therapy and diagnostics in a single platform, offer new possibilities for targeted drug delivery and real-time treatment monitoring in brain tumors. The review also addresses socioeconomic and demographic factors influencing brain tumor incidence and outcomes. It highlights the stark disparities in care access and survival rates among different racial and ethnic groups, emphasizing the urgent need for PPCM strategies to address these inequities. Challenges in implementing PPCM for brain tumors include the blood-brain barrier, which limits drug delivery, and the need for more extensive clinical trials to validate new approaches. The authors stress the importance of interdisciplinary collaboration and data sharing to advance the field, making the audience feel united and part of a larger team. While PPCM holds great promise, the review emphasizes that it should complement, not replace, population-level interventions and standard-of-care treatments. The authors advocate for a balanced approach that leverages cutting-edge personalized strategies while ensuring broad access to effective treatments. In conclusion, PPCM represents a powerful tool in the fight against brain tumors, offering the potential for more targeted, effective, and less toxic treatments. However, realizing its full potential will require ongoing research, clinical validation, and policy interactions to address disparities in care access.

## Introduction and background

The landscape of healthcare is currently in a state of urgent transformation across multiple domains. These changes encompass not just advancements in medicine [1,2] but also shifts in healthcare delivery systems [3-5] and evolving disease diagnostic methodologies [6-8]. They are driven by increasing health awareness among educated populations [9,10], the emergence of new disease outbreaks [11-13], and the adoption of Westernized lifestyles altering disease profiles globally [14-16]. While longevity has improved worldwide [17,18], this gain is counterbalanced by a population boom and unfavorable age demographics [19-21]. This list, while extensive, is not exhaustive [22,23], but it presents a complex set of healthcare challenges that demand immediate attention.

This evolving health landscape necessitates innovative approaches, such as precision population medicine (PPM), which holds the potential to revolutionize healthcare. PPM leverages genomic medicine, big data analysis, AI, wearable devices, and other digital technologies to enhance treatment outcomes. Healthcare leaders, including physician bodies, public health specialists, governing entities, political leaders, and the general population, recognize these challenges and seek comprehensive solutions. This series of reports aims to demonstrate how precision population cancer medicine (PPCM) and PPM [24,25] can contribute to improving cancer care (CC) outcomes universally, including the resource-scarce populations. 

Our aim is to educate and empower a diverse cohort of healthcare students, residents, fellows, and those interested in lifelong learning. We welcome individuals from interdisciplinary backgrounds, bridging medicine, nursing, public health, and policy. [26]. Our inclusive approach aims to prepare current and future healthcare professionals and other interdisciplinary students and researchers to contribute significantly to global health improvement and disease management [27]. The present report focuses on the continuum of brain neoplasms, covering benign to malignant classifications. We acknowledge the diverse expertise of our readers and solicit the academic community's forbearance for the varying levels of detail presented. This is the third report of our group’s series on PPM [24,25].

Spanning a spectrum from benign to malignant neoplasms, nervous system (NS) tumors include those originating in the brain, the spinal cord, and other peripheral nervous tissues [28]. Brain and central nervous system (CNS) tumors are the second leading cause of cancer among adolescents and young adults. Equally concerning fact is that they rank as the eighth most common cancer in older adults. This dual prominence is an alarming reality [29]. Despite sometimes being histopathologically innocuous, their precarious lodging in the constricted cranial/spinal confines contributes substantially to both mortality and morbidity [30,31]. As the third installment in our series, this work builds upon previous contributions to the evolving field of precision medicine (PM) [24,25]. In this intricate realm of neuro-oncology, brain tumors are the singular focus of this report. NS tumor statistics adapted from the Centers for Disease Control and Prevention (CDC) [32] and other prominent sources [33] are displayed in Table 1.

**Table 1:** Brain and other nervous system cancer incidence and mortality rates in the United States and Mississippi. This information from Centers for Disease Control and Prevention [32,33] is in the public domain and can be freely used or reproduced without obtaining copyright permission. * Mississippi data are included since the senior and corresponding author is from Mississippi, USA, and the data are from the rest of the USA.

Variable	United States (2017-2022)		Mississippi (2017-2022)*	
Type of race	Incidence	Mortality	Incidence	Mortality
Caucasians	87,257	71,665	706	154
African Americans	8,581	5,967	199	39
Other races	17,776	9,986	N/A	N/A
Total	113,614	87,618	905	193

Primary brain tumors exhibit varying prevalence between adults and children. The most common neoplasms in adults are glioblastoma multiforme (GBM), meningioma, and schwannoma. For the pediatric age group, the most frequent are pilocytic astrocytoma, ependymoma, and medulloblastoma. Secondary brain tumors, also known as metastatic tumors, typically originate from cancers outside the CNS before spreading to the brain. Table 2 exhibits the frequency of the most common brain metastases [34] and metastatic frequency to the spinal cord [35]. In 2024, brain tumors are projected to account for 25,400 new cancer cases, representing 1.3% of all new cancer diagnoses in the USA. Despite their relatively low incidence, brain tumors are expected to cause 18,760 deaths in the same year, comprising 3.1% of all cancer-related fatalities [33]. This is a result of their low five-year survival rate of just 33.4% [33,36], a stark reality that underscores the challenges in treating these tumors. Table 3 compares the five-year survival rate of brain and nervous system tumors to that of other prevalent tumors in the United States [37]. These rates have not substantially improved for several decades, although modest improvements have been observed in the past 15-20 years [38]. This is in part aided by the blood-brain barrier (BBB), which stands as an unwitting accomplice, shielding these tumors from potential pharmaceutical saviors [37].

**Table 2:** Five-year survival rates for prevalent cancers in the United States This tabulated information compares the five-year survival rates across various primary cancer sites, based on data compiled from Siegel et al. [37].

Tumor location	Five-year survival (%)
Brain, other nervous system	40
Breast	83
Colon, rectum	60
Larynx	53
Lung	21
Oral cavity	52
Prostate	97
Skin	70
Stomach	34
Thyroid	97

**Table 3:** Frequency of metastases to the brain and spinal cord. This tabulated information represents the frequency of brain and spinal cord metastases from various primary cancer sites, based on data compiled from Lauko et al. [34] and Ziu et al. [35].

Brain		Spinal cord	
Metastases	Frequency	Metastases	Frequency
Lung	20-56	Breast	21
Breast	5-20	Lung	19
Melanoma	7-16	Prostate	7.5

For decades, the war against primary brain tumors has been a series of Pyrrhic victories [38]. The infamous GBM, the most common malignant adult primary brain tumor, has a median survival time of eight to 15 months after diagnosis and a two- to three-year survival of about 4-7%. This, at least, in part, has been attributed to its highly proliferative nature and genetic heterogeneity [39,40]. However, a glimmer of hope shines through the National Program of Cancer Registries (NPCR) data, hinting at small but meaningful advances [38].

In this high-stakes clinical frontier, treatment strategies vary based on tumor type and location and are as varied as they are complex [36]. A novel approach integrates genomics, proteomics, and advanced computational methods, representing the vanguard of PM [25]. This innovative arsenal not only complements the traditional triad of surgery, radiation therapy (RT), and chemotherapy against the relentless neural siege [39] but also holds great promise. The PM-style treatment strategy shows promise, particularly as the most malignant brain tumors show extensive mutational instability that arises due to various genetic and molecular mutations, making them potentially targetable and responsive to these innovative therapies.

## Review

Charting the progress of brain tumor (GBM) therapy from the 1980s to today

**Figure 1:**
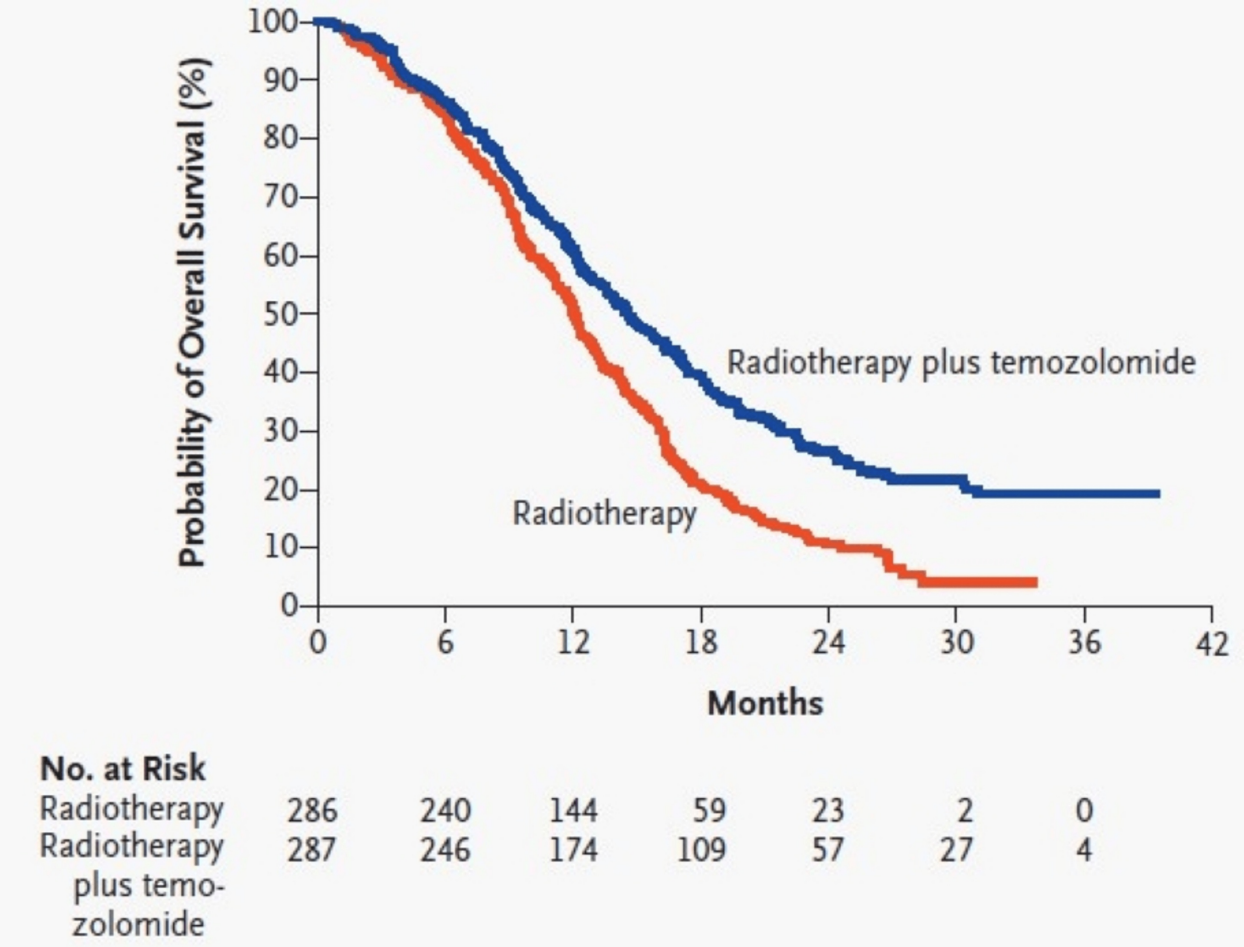
Kaplan–Meier estimates of overall survival according to the treatment group. This image is reproduced from Stupp et al. [42], and permission was obtained for the licensed content from the publisher, Massachusetts Medical Society.

**Figure 2:**
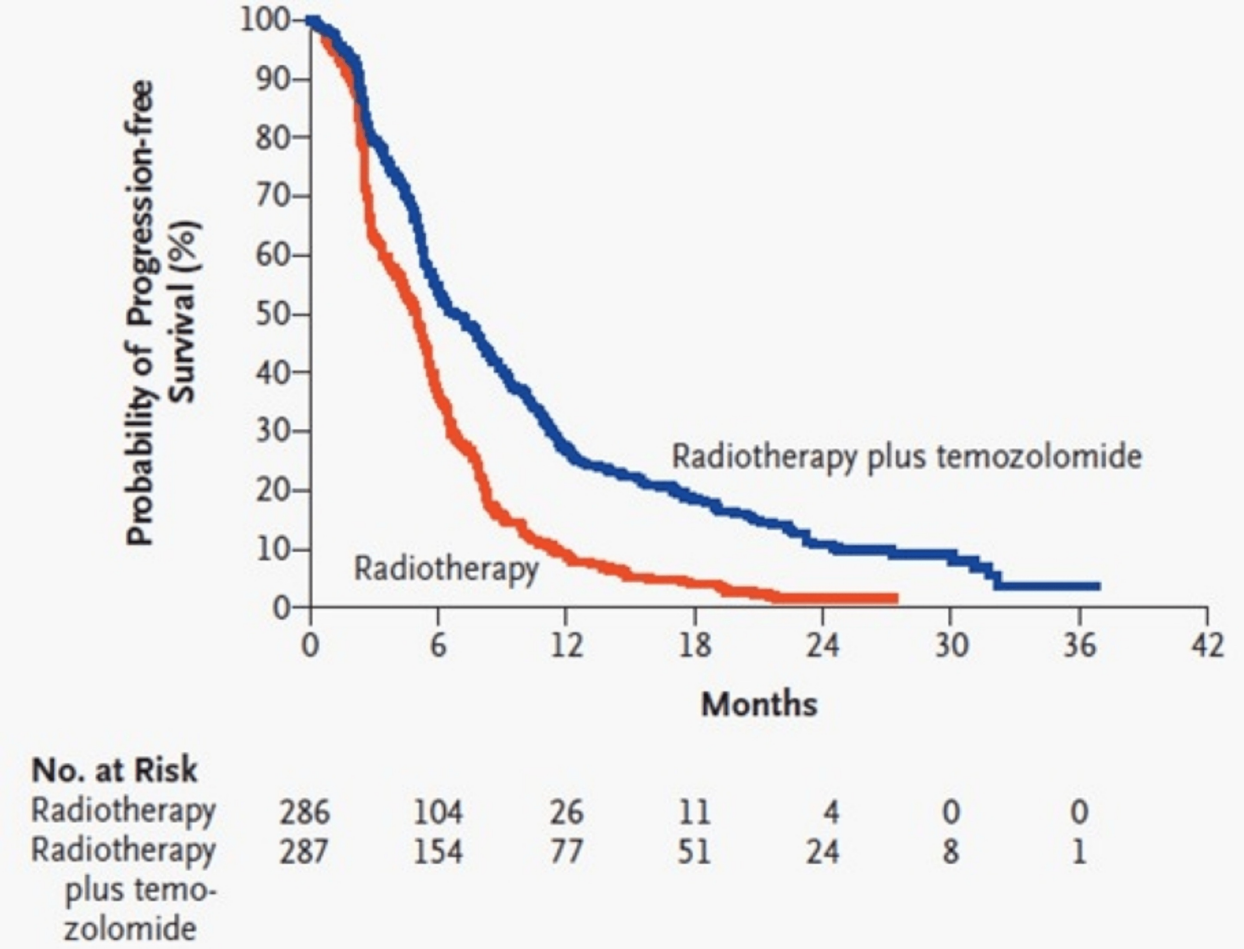
Kaplan–Meier estimates of progression-free survival according to the treatment group. This image is reproduced from Stupp et al. [42], and permission was obtained for the licensed content from the publisher, Massachusetts Medical Society.

For most of the post-World War II era, neurosurgeons operated in a world of limited imaging technology. Surgery was a delicate balance of skill and intuition. Meanwhile, early RT machines, being imprecise, bombarded tumors with brute force, often causing neurological sequelae. Little did anyone know that an imaging revolution was on the horizon. The advent of computerized tomographic (CT) scans in the 1970s and magnetic resonance imaging (MRI) in the 1980s transformed imaging capabilities [41]. This ongoing imaging renaissance, coupled with MRI-based functional imaging, image guidance-based RT planning, and neuronavigation systems, has armed modern neurosurgical, radiation, and neuro-oncologists with unparalleled precision to treat tumors, even in critical areas. The early 2000s witnessed temozolomide emerge as a crucial component of the treatment ensemble against brain tumors. When combined with RT, as outlined in the groundbreaking Stupp protocol, a specific treatment regimen developed by Dr. Roger Stupp [42], it led to significant improvements in survival rates (Figures 1, 2). This marked a turning point in GBM treatment - a practice-changing progress. Table 4 showcases the multi-modality advances made since the 1980s in the management of brain tumors in general, with a specific focus on GBM [43-45].

**Table 4:** Chronology of major advancements in brain tumor care, highlighting glioblastoma multiforme (1980-present) Abbreviations: MRI: magnetic resonance imaging, Gd: gadolinium, TMZ: temozolomide, 3-DCRT: three-dimensional conformal radiotherapy, IMRT: intensity-modulated radiotherapy, SRT: stereotactic radiotherapy, IGRT: image-guided radiotherapy, T1: spin-lattice relaxation time This tabulated information represents the diagnostic and treatment milestones for brain tumors, focusing specifically on glioblastoma multiforme, data compiled from multiple prominent sources: Castillo et al. [41], Stupp et al. [42], Vijayakumar et al. [43], Tsien et al. [44], and Thibouw et al. [45].

Advances in diagnosis and management	Approximate year(s)
Use of MRI in the diagnosis [41]. Note: The current standard of care is to use MRI-defined targets in radiotherapy	1984 – Use of MRI in brain. 1988 – Introduction of Gd, magnetic resonance spectroscopy. Early 1990’s – MRI in the operating room – (not routine in most places). 2008 – High-field MRI and still in development including potentially identifying molecular sub-types.
Modern chemoradiotherapy – Stupp’s regimen [42]: Fractionated daily radiotherapy, 60 Grays in 30 fractions over six weeks plus continuous daily oral TMZ (75 mg per square meter of body-surface area per day, seven days per week from the first to the last day of radiotherapy). After a four-week treatment-free break six cycles of adjuvant TMZ (150 to 200 mg per square meter for five days during each 28-day cycle.	2005 Stupp’s regimen [42] – Class I clinical results demonstrating median and two-year survival benefit: median survival (Figure 1) - treatment: 14.6 months vs. control: 12.1 months, two-year survival rate (Figure 1) - treatment: 26.5% vs. control: 10.4%
3-DCRT [43]	The 1990’s 3-DCRT dose escalation studies [44] laid the groundwork for future cutting-edge targeted radiation treatments.
IMRT, SRT, and IGRT	Since the 2000’s progressive steps from 3-DCRT TO IMRT TO IGRT [45] led to fewer complications and better quality of life; however, class 1 evidence of improved survival with these improved techniques is lacking.
Surgical technical improvements leading to higher maximal safe gross total resection of T1 Gd-weighted enhanced volume – a very important prognostic factor for better outcomes including survival.	Evolving practices that emerged since 2010 [46,47] - These improvements include (a) surgical navigational systems with functional MRI, (b) functional monitoring, (c) fluorescence-dye-visualization guided resection, (d) intraoperative MRI, (e) brain mapping in awake patients, (f) evoked potentials, (g) electromyography (EMG). The full cumulative benefits of these advances are still to be fully demonstrated with class 1 evidence convincingly; however, there are reports of very modest survival benefits.

**Figure 3:**
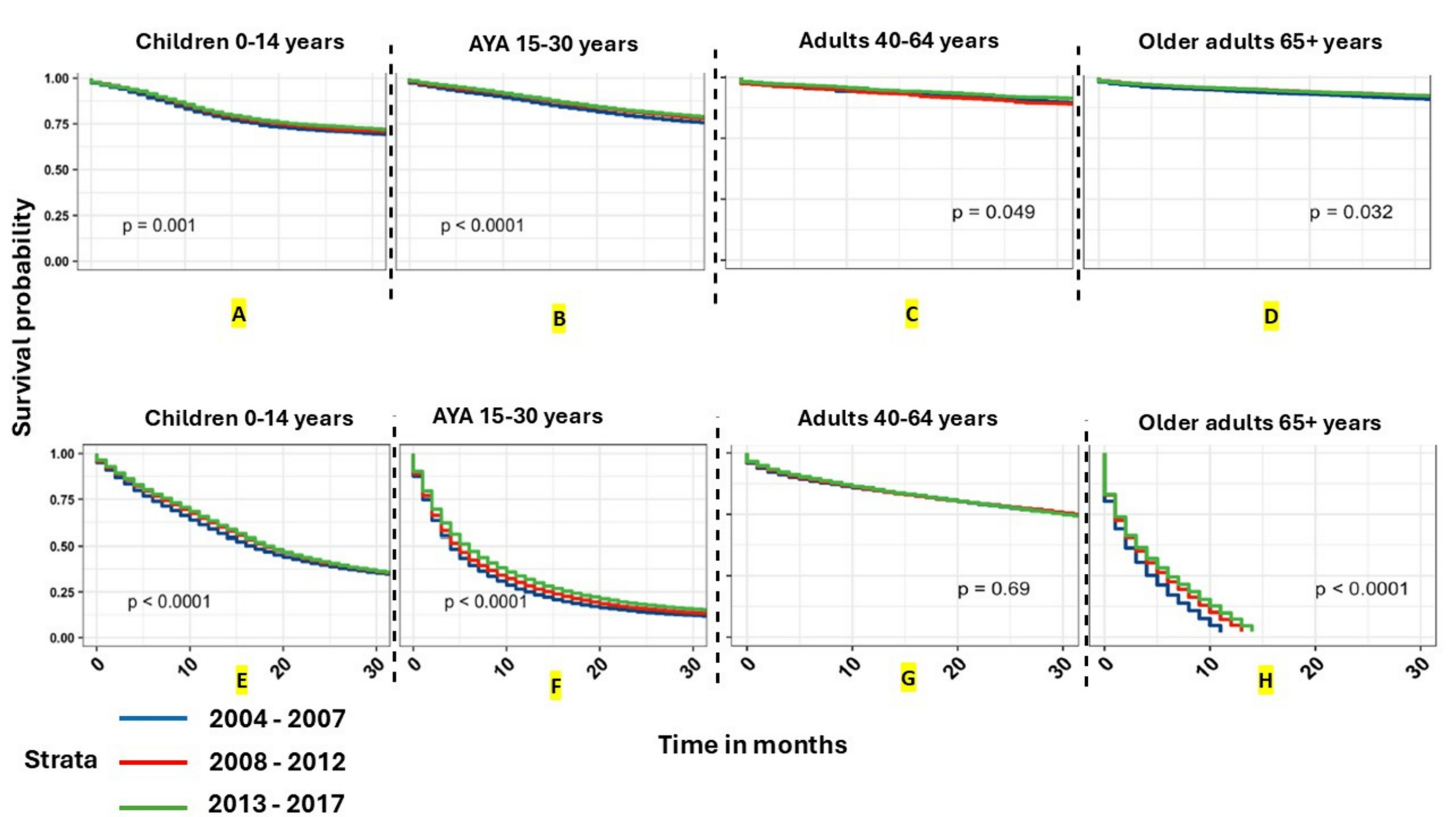
Graphs A through H depict overall survival curves stratified by age group. Kaplan-Meier overall survival curves for all primary brain and other CNS tumors classified by malignant behavior (A-D), stratified by age (group A: children 0-14 years, B: AYA 15-30 years, C: adults 40-64 years, and D: older adults 65+ years). The other sub-group is classified as non-malignant (E-H), stratified by age (group E: children 0-14 years, F: AYA 15-30 years, G: adults 40-64 years, and H: older adults 65+ years). The time period of diagnosis (2004–2007, 2008–2012, 2013–2017). P-values were determined by a log-rank test. (NPCR Survival Data: Data provided by CDC’s National Program of Cancer Registries SEER*Stat Database: NPCR Survival Analytic file, 2004–2017) Abbreviations: AYA: adolescents and young adults (age group 15-39 years), NPCR: National Program of Cancer Registries, CDC: Center for Disease Control and Prevention, SEER: Surveillance, Epidemiology and End Results, CNS: central nervous system Author's comments: (a) Although statistical improvement is seen after 2004-2007 years, they are not dramatically improved in terms of clinical significance. (b) It can be hypothesized that the more recent "statistical" improvements are likely from the PM advances since those advances became more clinically available after about 2010 as mentioned in Table 4. The image and caption are reproduced from Cioffi et al. [38] and available via Creative Commons Attribution 4.0 International License.

**Figure 4:**
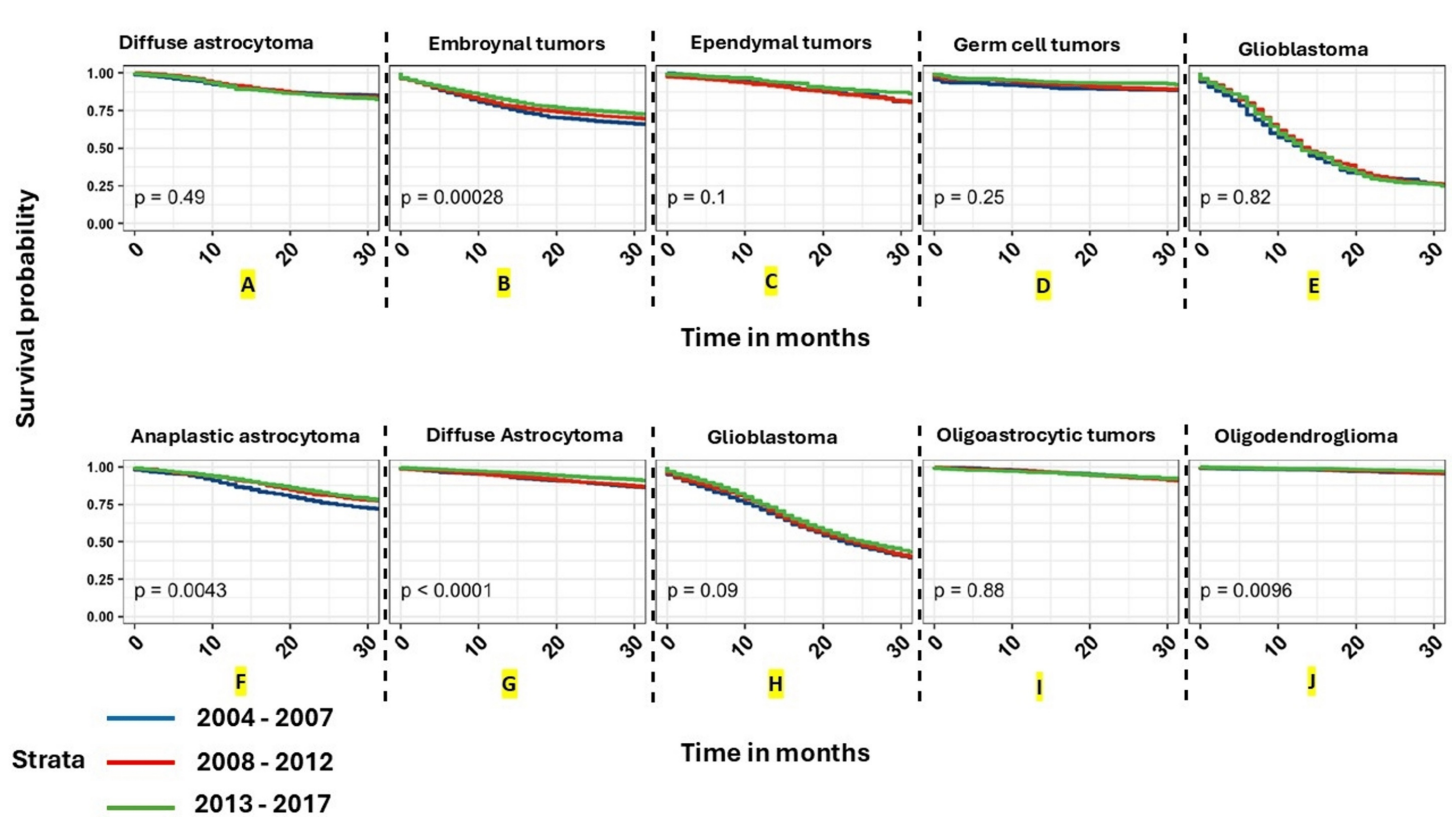
Graphs A through J depict overall survival curves for malignant brain and other CNS tumors. Kaplan-Meier overall survival curves for malignant brain and other CNS tumors stratified by year of diagnosis for the five most common histopathologies stratified by age group (children aged 0–14 years (A- E) (A- diffuse astrocytoma, B- embroynal tumors, C- ependymal tumors, D- germ cell tumors, E- glioblastoma) and AYA aged 15–39 years (F-J) (F- anaplastic astrocytoma, G- diffuse astrocytoma, H- glioblastoma, I- oligoastrocytic tumors, J- oligodendroglioma)). Time period of diagnosis (2004–2007, 2008–2012, 2013–2017). p-values were determined by a log-rank test. (NPCR Survival Data: Data provided by CDC’s National Program of Cancer Registries SEER Stat Database: NPCR Survival Analytic file, 2004–2017) Abbreviations: AYA: adolescents and young adults (age group 15-39 years), NPCR: National Program of Cancer Registries, CDC: Center for Disease Control and Prevention, SEER: Surveillance, Epidemiology and End Results, CNS: central nervous system The image and caption are reproduced from Cioffi et al. [38] and available via Creative Commons Attribution 4.0 International License.

**Figure 5:**
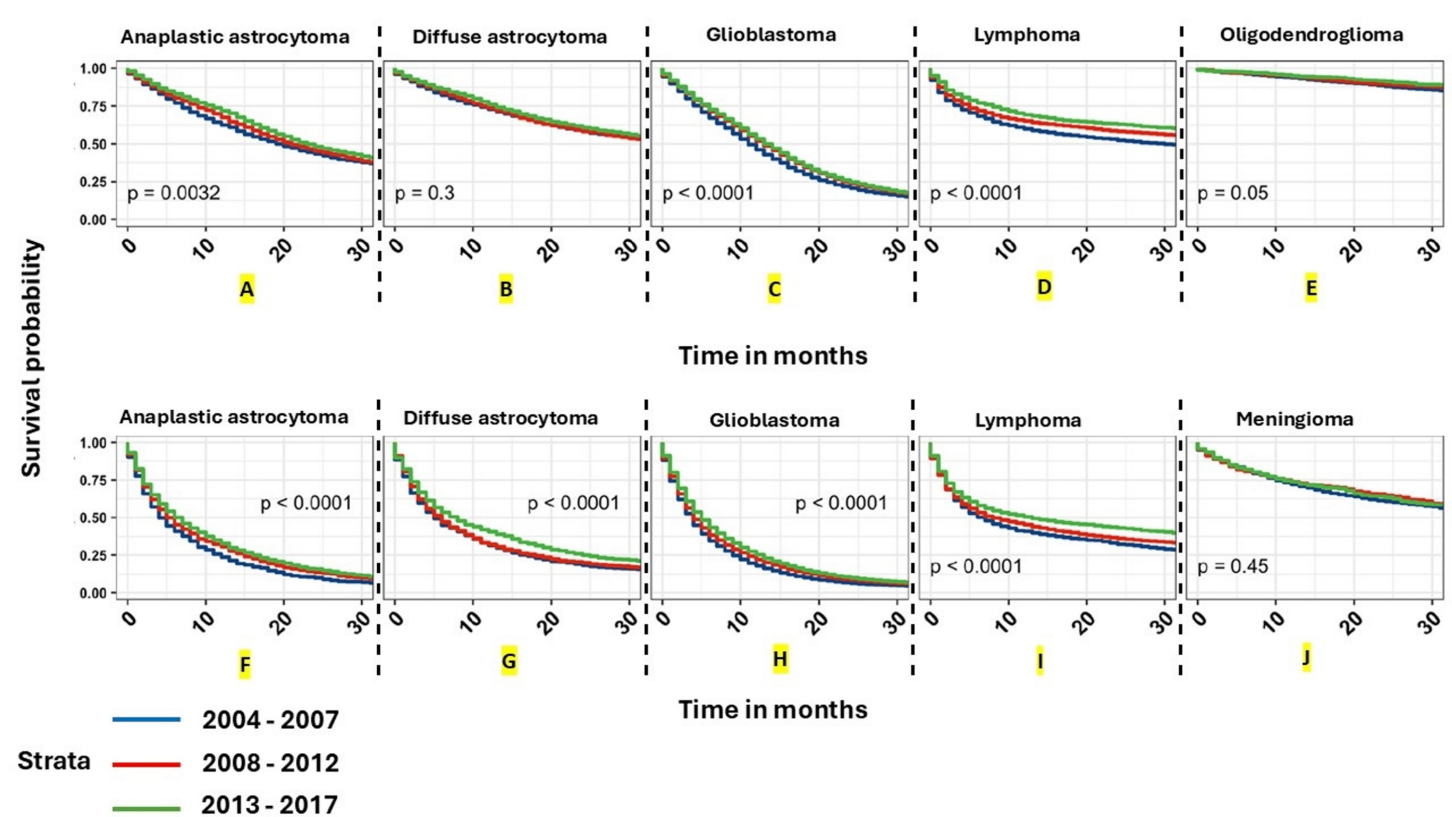
Graphs A through J depict overall survival curves for malignant brain and other CNS tumors Kaplan-Meier overall survival curves for malignant brain and other CNS tumors stratified by year of diagnosis for the five most common histopathologies stratified by age group (adults aged 40–64 years (A-E) (A- anaplastic astrocytoma, B- diffuse astrocytoma, C- glioblastoma, D- lymphoma, E- oligodendroglioma) and older adults aged 65 + years (F-J) (F- anaplastic astrocytoma, G: diffuse astrocytoma, H: glioblastoma, I: lymphoma, J: meningioma)). Time period of diagnosis (2004– 2007, 2008–2012, 2013–2017). p-values were determined by a log-rank test. (NPCR Survival Data: Data provided by CDC’s National Program of Cancer Registries SEER Stat Database: NPCR Survival Analytic file, 2004–2017) Abbreviations: NPCR: National Program of Cancer Registries, CDC: Center for Disease Control and Prevention, SEER: Surveillance, Epidemiology and End Results, CNS: central nervous system The image and caption are reproduced from Cioffi et al. [38] and available via Creative Commons Attribution 4.0 International License.

**Figure 6:**
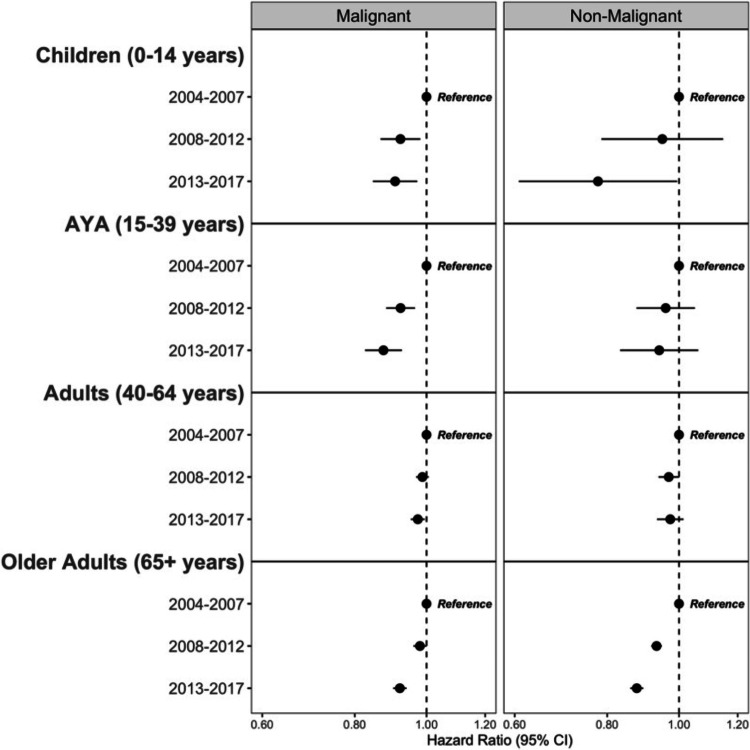
Multivariable Cox proportional hazards forest plots. Multivariable Cox proportional hazards forest plots comparing overall survival by time period of diagnosis for primary brain and other CNS tumors stratified by behavior and age group. All models adjusted for sex, race/ethnicity, and treatment pattern. (NPCR Survival Data: Data provided by CDC’s National Program of Cancer Registries SEER*Stat Database: NPCR Survival Analytic file, 2004–2017) Abbreviations: AYA - Adolescents and young adults (age Group 15-39 years), NPCR - National Program of Cancer Registries, CDC - Center for Disease Control and Prevention, SEER - Surveillance, Epidemiology and End Results, CNS - Central nervous system The image and caption are reproduced from Cioffi et al. [38] and available via Creative Commons Attribution 4.0 International License.

The above-tabulated advances in the multimodality management of brain tumors have led to improvements in outcomes, albeit with varying degrees of success across different tumor types and patient groups. The outcome improvements are more substantial for pediatric / adolescents and young adults (AYA) groups than for adults. However, for patients with GBM, there has only been a modest increase in survival. The study by Cioffi et al. [38] substantiates this by reporting the data from tumor registries - thus representing academic and community settings. The results are depicted in Figures 3-6.

**Figure 7:**
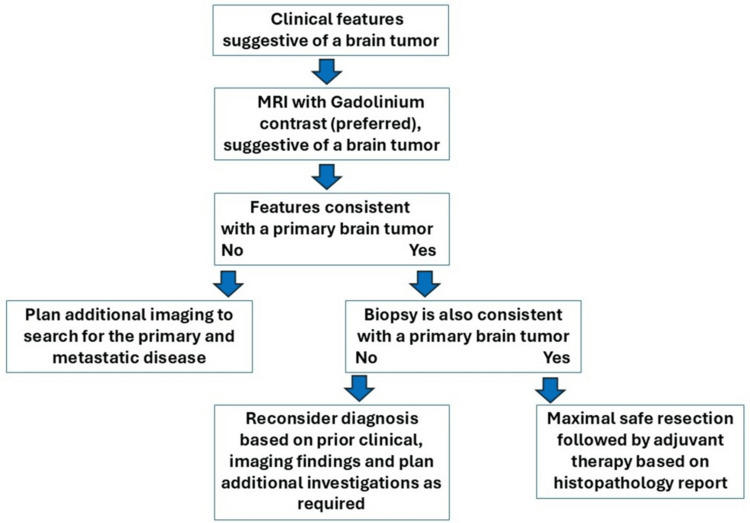
Algorithm to detect and treat primary brain tumors. Abbreviation: MRI: magnetic resonance imaging This flowchart illustrates the recommended diagnostic and treatment pathway for brain tumors, integrating guidelines from Perkins et al. [36] and Wong [48].

The management of primary brain tumors in adults requires a multidisciplinary approach [48], integrating various diagnostic and therapeutic strategies. To illustrate this complex process, we present a comprehensive algorithm based on current best practices The algorithm shown in Figure 7, reworked from Perkins et al. [36] and other prominent sources [48], outlines the detection and treatment pathways of primary brain tumors in adults. Due to the difficulty of diagnosing brain tumors, similar treatment plans will become useful after including additional factors, such as patient-specific details, epigenetics, and other components of PPM, and thus will further personalize diagnostic and management strategies in the future.

The goal of our report is to highlight recent advancements in PM that have the potential to significantly enhance future outcomes. In addition, we aim to adopt a personalized PPM strategy to create a more comprehensive interdisciplinary approach. This approach is designed to yield quicker benefits and ensure that disadvantaged populations are not overlooked, fostering a sense of empathy and consideration in our readers.

Social factors related to brain tumors

Socioeconomic Factors

In the nuanced realm of brain tumors, a complex tapestry of socioeconomic factors like age, race, ethnicity, and living conditions weave their influence on patient survival. The harsh reality for patients in the lowest socioeconomic quartile with diminished access to healthcare means missed opportunities for life-saving treatments like chemotherapy, radiation, or surgery. They are thus faced with significantly worse overall survival rates [49].

For glioma patients, the narratives take an unexpected turn. It is not always the case that the higher the socioeconomic status (SES), the better the outcomes. This intriguing twist in the story keeps the audience engaged and eager to learn more. From 2000 to 2010, the incidence of glioma and glioblastoma in affluent US counties was -1.14 times higher than their less affluent counterparts, as reported by the Surveillance, Epidemiology, and End Results (SEER) program [50]. A similar trend emerges for intracranial meningiomas, likely driven by more frequent tumor detection in high SES populations rather than an actual increase in occurrence [51]. While environmental factors have been suggested as contributors to this phenomenon, no definitive link has been established [51]. As part of the PPM approach, which involves tailoring medical treatment to the individual characteristics of each patient, confirming the impact of these factors could revolutionize patient therapy recommendations. This could potentially target lifestyle adjustments (e.g., cell phone use or smoking habits, if such factors show any etiological influence).

Interestingly, medulloblastomas also display a perplexing correlation between lower education levels and improved survival rates [52]. These correlations are difficult to explain since most risk factors regarding SES and survival/incidence rates, such as access to screening and treatment and prevalence of infectious disease, all seem to fade into the background when it comes to gliomas and glioblastomas [53]. Unlike other cancers, no known occupational hazards or exposure risks explain their formation [50]. Further exploration into the origins of these tumors and their elusive etiology using the PPM strategies explained in our first and second papers of this series [24,25], which delve into the application of PM in oncology, can potentially lead to more effectively delivering PPM-based care to affected patients. Such approaches, we hope, will lead to an improvement in outcomes. *Racial Disparities*

**Figure 8:**
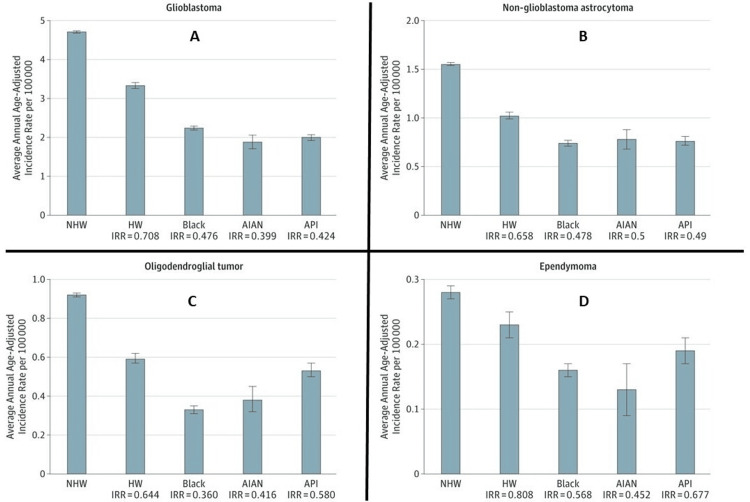
Graphs A-D represent the annual incidence rates of different brain tumor types across ethnic groups. The graphs A, B, C, and D represent the annual incidence rates across ethnic groups of glioblastoma, non-glioblastoma astrocytoma, oligodendroglial tumor, and ependymoma, respectively. Abbreviations: NHW: non-Hispanic White, HW: Hispanic White , AIAN: American Indian and Alaska Native, API: Asian and Pacific Islander, IRR: incidence rate ratio The image is reproduced from Ostrom et al. [54] and permission was obtained for the licensed content from the publisher, American Medical Association.

Brain tumor survival rates present a complex puzzle, with racial and ethnic factors adding layers of intricacy. While it is often stated that African Americans and Hispanics face poorer outcomes compared to Caucasians, this broad-brush approach can inadvertently perpetuate disparities and fail to serve patients effectively. Delving deeper into the data reveals a more nuanced picture. Ostrom et al. found that non-Hispanic White people in the United States paradoxically possess both the highest incidence rates of glioblastoma and the lowest five-year relative survival rate, a mere 4.8% compared to ​8.8% among Asians or Pacific Islanders [54]. This seemingly contradictory finding underscores the intricate nature of brain tumor biology and epidemiology, challenging us to unravel its complexities. Figure 8, adapted from a study by Ostrom et al. [54], is a visual representation of the annual incidence rates of different brain tumor types across ethnic groups, including non-Hispanic Whites, Hispanic Whites, Blacks, Asian or Pacific Islanders, and American Indians. It illustrates that non-Hispanic Whites have notably higher frequencies of brain tumor diagnoses compared to other ethnic populations.

What multifaceted influences shape these racial inequalities? The culprits are likely manifold: a) differences in tumor biology and normal immune responses, b) environmental factors, c) potential access to care issues, and d) care-provider-based unconscious biases. Communication issues and patient/family disease education barriers with care providers can create chasms of misunderstanding. Brown et al. point out that systemic and systematic issues can permeate every facet of care; their influence can be pervasive and profound [55]. However, hope is not lost in this labyrinth of disparities. A potential pathway forward unfolds in the form of shared decision-making. By inviting patients and families to participate actively in their care management, physicians can bridge cultural divides, dismantle barriers to treatment, and gain invaluable insights into each patient's unique background [56]. This collaborative approach promises to chip away at the monolith of racial disparities, one patient at a time, as we strive for a future where survival rates know no color.

Sex and Age

Sex differences in brain tumor epidemiology are not just a mere observation, but a significant factor that piques our interest. Men, across most brain tumor types, exhibit higher incidence rates, a trend that persists across racial and ethnic groups. Notably, GBM shows increased male incidence rates across all ethnicities [57]. The X chromosome is hypothesized to contribute to tumorigenesis, potentially influencing male predisposition to astrocyte tumor development. While various theories attempt to explain this phenomenon, a definitive cause remains elusive. A study by Yang et al. [58], utilizing MRI-based imaging analysis, revealed that standard treatment (utilizing TMZ chemotherapy) for GBM demonstrates greater efficacy in women than men. A promising avenue for addressing this disparity may lie in targeted therapy against the Isocitrate dehydrogenase 1(IDH1) mutation and sex-specific gene clusters [58].

Age also factors significantly in brain tumor incidence. While most types show increased occurrence with age, malignant gliomas present a distinctive bimodal distribution. Individuals over 60 years old demonstrate the highest incidence rates for common brain tumors such as GBM, vestibular schwannoma, and meningioma, although the etiology is likely multifactorial [59]. Currently, only ionizing radiation and genetic predisposition are definitively linked to brain tumor formation. Other potential risk factors, including non-ionizing radiation, smoking, and electromagnetic field exposure, remain under investigation.

In this evolving narrative of medical science, each new discovery brings us closer to understanding the complex interplay of factors that shape brain tumor incidence and outcomes. In its quest to optimize patient care, PPM [24,25] broadens its scope to encompass social factors, socioeconomic conditions, racial disparities, and demographics, necessitating a deeper delve into the root causes of these disparities. This narrative of human health and scientific inquiry remains compelling and unfinished.

Genetic factors in the treatment of brain tumors

Challenges of Extensive Heterogeneity

The genetic underpinnings of brain tumor development and progression are not just complex, but they mirror the broader concept of genetic etiologies in tumorigenesis. A significant challenge in genetically characterizing these tumors lies in their remarkable heterogeneity. Within a single tumor, cells can exhibit varying grades and extensive genetic diversity, complicating the development of targeted therapies [60]. This complexity is what keeps us intrigued and challenged in our quest to understand and treat brain tumors.

**Figure 9:**
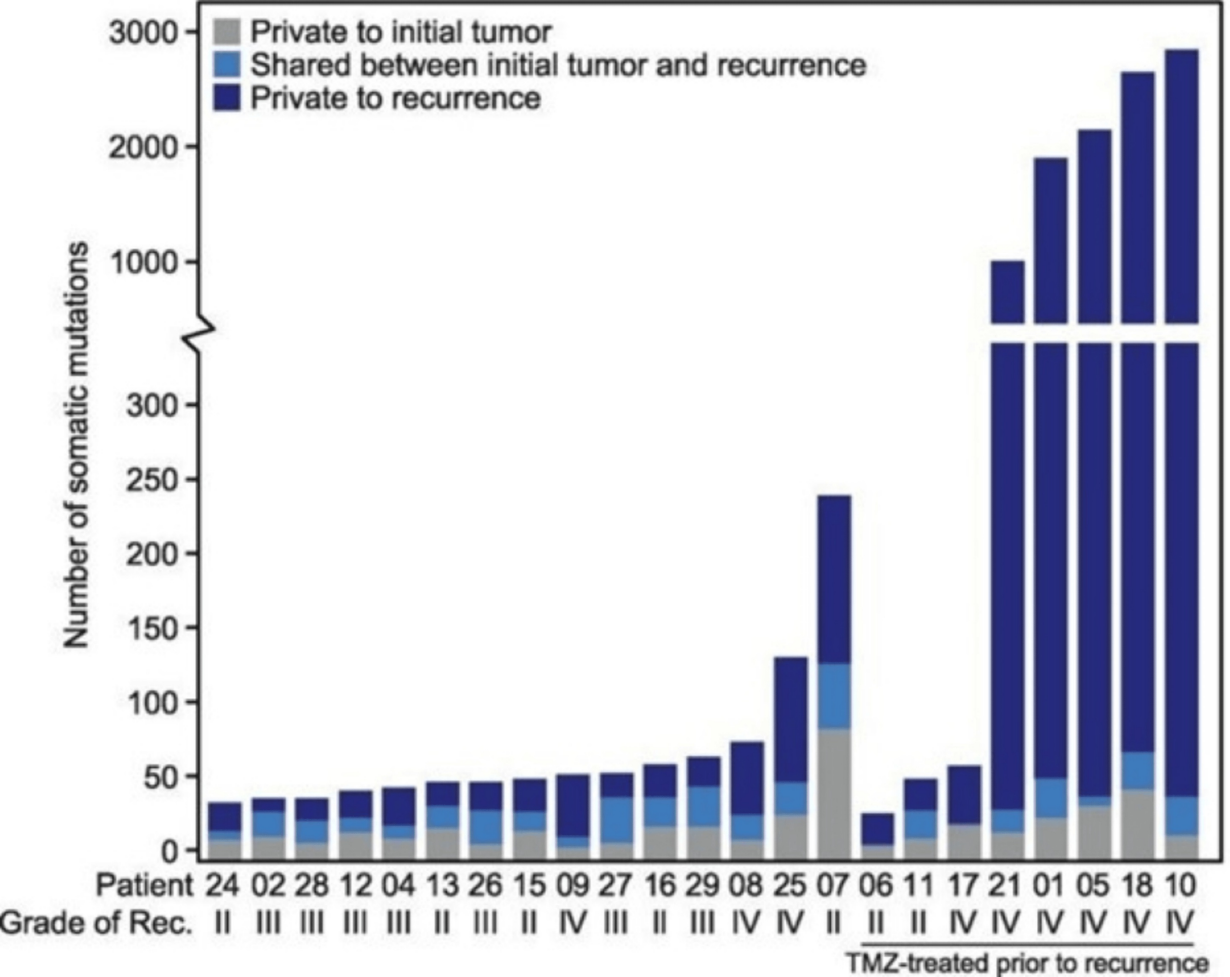
Unique and shared mutations between the index and recurrent glioma. Abbreviations: TMZ: temozolomide, Rec.: recurrence The image is reproduced from Johnson et al [61] and permission was obtained for the licensed content from the publisher, The American Association for the Advancement of Science.

Recurrent tumors often display even more significant genetic variability than their initial counterparts, sometimes through alternative driver mutations. As illustrated in Figure 9, a study by Johnson et al. [61] revealed that in 43% of cases, most mutations present in the initial glioma tumors were absent in recurrences. The figure shows the relative number of mutations unique to and shared by initial and recurrent glioma in the patients studied. There is a vast amount of heterogeneity between initial and recurrent tumors, making it difficult to treat the initial and recurrent tumors with similar strategies. In addition, TMZ treatment was associated with exceptionally high levels of mutation in grade IV tumors, suggesting a correlation with tumor hypermutation in these patients. This suggests that recurrent tumors, originating from early-stage cells of the primary tumor, likely expand their genetic diversity in parallel with the initial tumor's development [61]. Intriguingly, driver mutations in GBM may be lost upon recurrence, despite their presence in various cell populations of the initial tumor [60]. This phenomenon is also observed in pediatric populations, where recurrent GBM tumors may appear similar to the initial tumor macroscopically but differ significantly at the molecular level. Notably, these recurrent tumors often exhibit increased amplification of the MYCN gene [62].

Genetic Patterns Associated With IDH1 Mutations

The investigation of genetic signatures linked to IDH1 mutations provides valuable perspectives on their significance in glioma biology. IDH1 plays a crucial role in the citric acid cycle, catalyzing the formation of 2-hydroxyglutarate (2-HG). The most prevalent IDH1 mutation, a substitution at R132 (typically R132H), occurs in the enzyme's active site, disrupting 2-HG production [63,64]. This mutation is frequently observed in various brain tumors, including 88% of low-grade diffuse astrocytomas, 82% of secondary glioblastomas, 79% of oligodendrogliomas, and 94% of oligoastrocytomas [63]. Recently, IDH1 mutations have become targets for monoclonal antibody therapies [65,66]. However, their prevalence in only 12% of total GBM cases may limit their therapeutic potential primarily in low-grade gliomas64. Interestingly, while IDH1 mutations may be lost in recurrent GBM [60], they often persist in recurrent gliomas and may be the sole initial mutation driving the recurrence [61].

IDH1 mutations are associated with other genetic alterations involved in glioma pathogenesis and progression. For instance, tumor protein p53 (TP53) mutations co-occur with IDH1 mutations in up to 63% of low-grade diffuse astrocytomas [63] and are linked to pediatric and adult glioblastomas [60-62]. Secondary glioblastomas often exhibit IDH1 and TP53 mutations, along with loss of heterozygosity at 10q25-qter and 22q12.3 [67,68]. By contrast, primary glioblastomas are more frequently associated with mutations in the epidermal growth factor receptor (EGFR), mouse double minute 2 homolog (MDM2), and PTEN, as well as loss of heterozygosity on chromosome 10p [67,69]. Evidence suggests that IDH1 mutations precede TP53 mutations when they co-occur [64]. In addition, alpha thalassemia/mental retardation syndrome X-linked (ATRX) mutations may follow a similar pattern in diffuse astrocytomas, while telomerase reverse transcriptase (TERT)-promoter mutations and 1p19q deletions in conjunction with IDH1 mutations are associated with oligodendrogliomas [70,71]. IDH1 mutations are also frequently accompanied by loss of heterozygosity in oligodendrogliomas [63]. Furthermore, these mutations are linked to epigenetic changes, particularly hypermethylation of multiple genes and histones (H3K9me2, H3K27me3, and H3K36me3). Through these mechanisms, IDH1 mutations are associated with the CpG island methylator phenotype (CIMP), a subset characterized by a distinct biological framework and extensive epigenetic profile [64,72].

Innovations in Targeted Treatments

The pivotal role of IDH1 mutations in brain tumors has made them a prime target for anti-tumor therapies. This focus on targeted therapies brings a sense of hope and optimism. A breakthrough came in 2013 with the development of a drug explicitly inhibiting R132H mutated IDH1 while sparing the wild-type form [65,73]. Subsequent advancements led to vorasidenib, a dual IDH1 and IDH2 inhibitor, which demonstrated favorable safety in a phase I glioma cohort [66]. Vorasidenib has shown promising results in grade 2 gliomas, improving progression-free survival (median progression-free survival of 27.7 months versus 11.1 months). The hazard ratio for disease progression or death in this study was 0.39, with 95% confidence interval (CI) of 0.27 to 0.56 and P < 0.001). In addition, the time to the next intervention was significantly extended in the vorasidenib group compared to the placebo group, albeit with increased adverse effects compared to placebo [74]. Compared to ivosidenib, another IDH1 and IDH2 inhibitor, vorasidenib, exhibited superior brain penetrance and more consistent 2-HG suppression, advancing to phase 3 testing [75].

Recent rodent studies suggest that combining these small-molecule inhibitors with current standard treatments - ionizing radiation, temozolomide, and a PD-L1 inhibitor - may offer optimal therapy for gliomas [71]. Targeting IDH1 exemplifies the progress made in genomic medicine, contributing to PM-based care for BT patients. While this approach efficiently addresses a large subset of glioma patients, it does not apply to BT without IDH1 mutations. Wild-type IDH gliomas are generally more aggressive and often associated with poorer prognoses. Future genetic/molecular biology research should focus on identifying commonalities in these tumors beyond IDH1 mutations. This approach could uncover new therapeutic targets and strategies.

Epigenetic Modifications as Causative Factors of Brain Tumors

**Figure 10:**
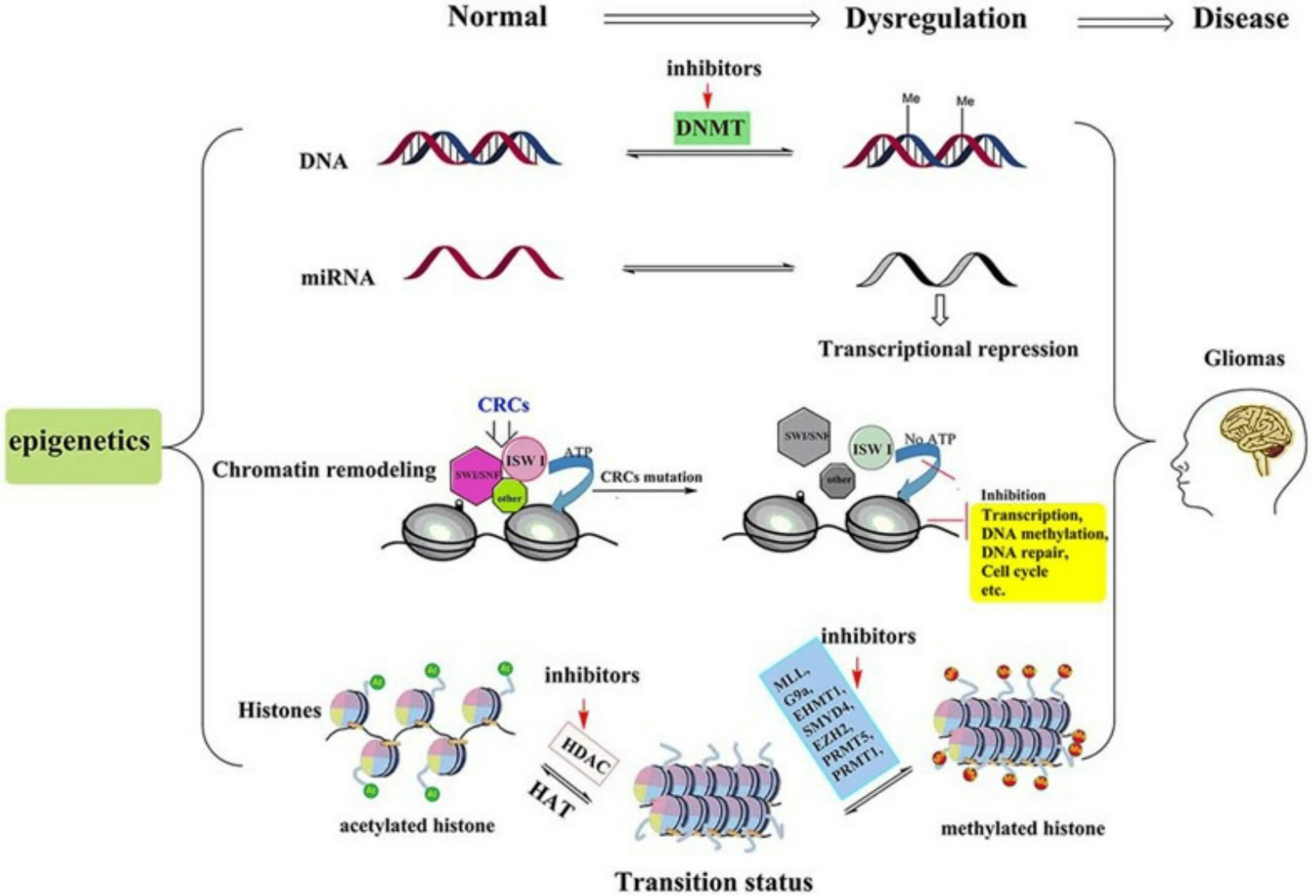
Key epigenetic modifications in gliomas. Abbreviations : DNA: deoxyribonucleic acid, miRNA: microRibonucleic acid methylation, DNMT: DNA methyltransferase, CRC: chromatin remodeling complexes, ATP: adenosine triphosphate, SWI/SNF: switch/sucrose non-fermentable, ISWI: imitation switch, HDAC: histone deacetylase, HAT: histone acetyltransferase , MLL: mixed-lineage leukemia, EHMT1: euchromatic histone-lysine N-methyltransferase 1, G9a/EHMT2: euchromatic histone-lysine N-methyltransferase 2, SMYD4: Su(var)3-9, enhancer-of-zeste and trithorax (SET) and myeloid, nervy, and DEAF-1 (MYND) domain containing 4, EZH2: enhancer of Zeste homolog 2, PRMT5: protein arginine methyltransferase 5, PRMT1: protein arginine methyltransferase 1 *​​​​​​​* *The image is reproduced from Zang et al. [77] and available via Creative Commons Attribution 4.0 International License.*

Epigenetic modifications, including DNA methylation, histone modifications, and non-coding RNA (ncRNA), play a significant role in the NS tumorigenesis [76]. Figure 10 from a study by Zang et al. [77] depicts the four essential epigenetic modifications that play crucial roles in glioma pathogenesis and serve as potential treatment targets. A prime example is DNA methyltransferase (DNMT), which can be targeted therapeutically using specific inhibitors. These DNMT inhibitors effectively mimic the methylation of the DNMT gene, thereby suppressing its expression and impeding glioma development and progression. This approach exemplifies how understanding epigenetic mechanisms can lead to novel therapeutic strategies in brain tumor treatment. Epigenetic alterations associated with DNA methyltransferase and gliomas regulate genome expression without changing the underlying DNA sequence [77]. The reversible nature of these changes has sparked increased interest in studying epigenetics to understand pathogenesis, develop treatment approaches, and identify novel detection and therapeutic strategies for brain tumors.

GBM is an illustrative example of epigenetic influences. The gene encoding O6-methylguanine-DNA methyltransferase (MGMT), a DNA repair enzyme, has emerged as a potential molecular biomarker for both malignancy detection and predicting the efficacy of adjuvant chemoradiotherapy [78]. Methylation of the MGMT gene promoter, resulting in gene silencing, serves as a predictive biomarker for radiation response and an independent prognostic factor for disease progression and overall survival. Studies have shown that the two-year survival rate nearly doubles for GBM patients with methylated MGMT compared to those with unmethylated MGMT (30% vs. 16%, respectively) [79]. Furthermore, epigenetic modifications are increasingly recognized as potential causative factors in GBM development [80].

Diagnosis and prognosis of brain tumors: the challenge of biomarkers

The development of epigenetic biomarker screening and diagnostic tests for gliomas and other brain tumors is hindered by the extensive tumor heterogeneity, which results in aberrant gene expression. This complexity limits the effectiveness of PM in providing efficient molecular diagnostics for larger patient populations [81]. When considering confirmatory diagnostic tools, weighing the advantages and disadvantages of tissue biopsies against their scalability for public use is crucial. Currently, patients with high clinical suspicion and suggestive imaging undergo tissue biopsy with pathological assessment to guide management. While brain tissue biopsy remains the primary diagnostic method, it carries potential neurological risks such as swelling and hemorrhage [82,83]. In addition, tissue biopsies may suffer from heterogeneous sampling "error" issues [84]. By contrast, liquid biopsy represents a non-invasive alternative, and it presents potentially significant advantages over invasive tissue biopsy for epigenetic studies, diagnostics, and prognostic techniques in brain tumors. While liquid biopsy for brain tumor epigenetics is currently the subject of extensive research, its clinical application remains limited compared to other established techniques [83,84], perhaps hindered by the blood-brain barrier (BBB). This approach aims to identify specific epigenetic signatures that could detect various primary and metastatic brain tumors and stratify outcomes at different points throughout a patient's clinical course [82-85]. Table 5 summarizes the findings of the studies by Saenz-Antoñanzas [83] and Becker [84].

**Table 5:** Tissue vs. liquid biopsy: comparing approaches in glioblastoma multiforme detection This tabulated information on tissue vs. liquid biopsy is based on data compiled from Saenz-Antoñanzas et al. [83] and Becker et al. [84].

	Invasiveness	Sampling accuracy	Scalability for population	Standard of practice
Tissue biopsy	More invasive	Heterogenous sampling (poor detection)	Less scalable (suspected patients)	Gold standard
Liquid biopsy	Less invasive (i.e. blood sample)	More representative of tumor composition	More scalable (all patients)	In development

Liquid biopsy enables the isolation of circulating tumor DNA (ctDNA) and cell-free DNA (cfDNA) to detect epigenetic alterations. A study examining promoter methylation profiles of MGMT, RASSF1A, p15INK4B, and p14ARF genes via serum cfDNA PCR testing demonstrated high concordance with paired biopsy samples, validating the liquid biopsy approach and encouraging further clinical biomarker research [86]. This technique effectively differentiated primary from metastatic brain tumors through cfDNA analysis of Ras association domain family member 1, isoform A (RASSF1A) methylation status. MicroRNAs (miRNAs), post-transcriptional regulators of gene expression in tumorigenesis, have also shown promise in liquid biopsies due to their stability in blood. For instance, miR-145-5p was identified as a reliable diagnostic marker for GBM in serum and served as an independent prognostic indicator for overall survival [87].

**Figure 11:**
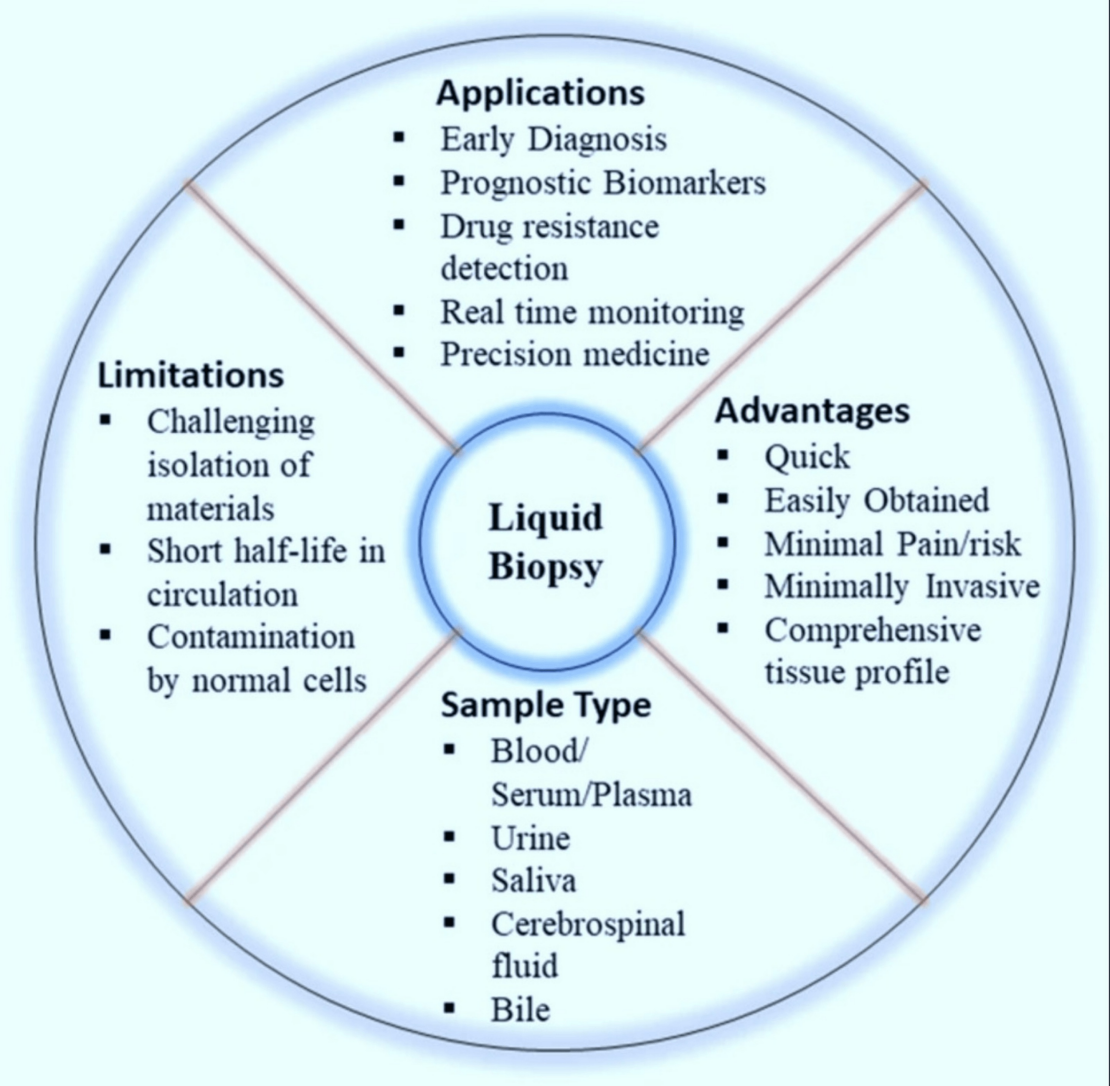
Liquid biopsy - applications, strengths, and challenges The image is reproduced from Preethi et al. [88], and available via Creative Commons Attribution 4.0 International License.

These reversible, tissue-specific epigenetic changes, controlled by gene-environment interactions, offer promising opportunities for detecting and treating brain tumors through a targeted PM approach. The non-invasive nature and potential for dynamic monitoring make liquid biopsy a valuable tool in the evolving landscape of brain tumor management. Figure 11, adapted with permission from the study by Preethi et al. [88], provides a visual summary of liquid biopsy in personalized brain tumor care. However, the widespread implementation of liquid biopsy in clinical practice requires an urgent increase in interventional clinical trials. Major consortia like the European Liquid Biopsy Society [89] in Europe and BLOODPAC [90] in the USA are spearheading significant programs for standardization of liquid biopsy results across different clinical settings by developing and validating a broad array of standard operating procedures [91]. Achieving this level of standardization will pave the way for liquid biopsy to become a routine and trusted tool, especially for tumors where no screening is available [92]. Such standardization can also help the use of liquid biopsies in the Global South nations.

Epigenetic Modifications as Therapeutic Targets

Despite the significant challenge presented by the BBB in brain tumor treatment, the potential of targeting epigenetic modifications offers a promising and hopeful approach to overcome this obstacle [81]. While the development and cost of novel therapeutics remain substantial barriers, the identification of numerous potential epigenetic targets for various brain tumors is a beacon of hope. To expedite brain tumor treatment, researchers are exploring the repurposing of existing drugs with known BBB penetration and CNS activity. For instance, disulfiram, an inexpensive medication used for chronic alcoholism, has demonstrated activity against GBM through MGMT inhibition in preclinical studies [93-95]. Deletion of lysine (K)-specific demethylase (KDM) 6B has been shown to enhance various immune pro-inflammatory pathways in the GBM tumor microenvironment, including interferon response, antigen presentation, and phagocytosis. This suggests that inhibiting KDM6B could potentially overcome myeloid-derived immune suppression and improve the efficacy of immunotherapy in GBM [96].

In the realm of bromo- and extra-terminal domain (BET) inhibitors, CC-90010 has emerged as a promising next-generation compound, encouraging antitumor activity in patients with advanced solid tumors [97]. This is particularly relevant for GBM, where bromodomain-containing protein 2 (BRD2) and BRD4 proteins are significantly overexpressed. Treatment with the BET protein inhibitor I-BET151 has been shown to inhibit GBM cell proliferation [98]. Furthermore, another BET inhibitor, OTX015, has demonstrated superior antiproliferative effects compared to its analog JQ1 in GBM cell lines [99]. Moreover, BT with IDH1/2 mutations is suitable for histone deacetylase (HDAC)-targeted therapy [100]. HDAC6-selective inhibitor ricolinostat (ACY-1215) significantly inhibited GBM cell growth [101]. These findings collectively highlight the potential of targeting epigenetic regulators, as therapeutic strategies for GBM, potentially enhancing both direct antitumor effects and immune-mediated responses.

Although epigenetic modifications have emerged as potential targets at various stages of brain tumor management the heterogeneous nature of BT complicates the prioritization of known epigenetic alterations [102]. The promising use of biomarker assays in liquid biopsy for diagnostics, along with emerging and repurposed pharmaceuticals, suggests that PM approaches can be broadly applied to populations. These advancements offer scalable and cost-effective treatment options for various brain tumors. As technological innovations progress, the potential to improve care and outcomes for underserved and disproportionately impacted populations becomes increasingly feasible. This evolving landscape of epigenetic-based therapies and diagnostics holds promise for more effective and accessible brain tumor management strategies, instilling a sense of optimism and hope for the future of brain tumor management.

Big data, artificial intelligence, imaging, and therapeutics

Big data, encompassing vast datasets including genomic, proteomic, metabolomic, clinical, epidemiological, and survival information, is poised to play a crucial role in advancing personalized medicine [103,104]. This is particularly relevant in the field of oncology, where the complex nature of cancer necessitates a personalized approach to treatment. A significant initiative in this direction is the Children's Brain Tumor Tissue Consortium, developed by Adam Resnick and colleagues from 13 US hospitals, including Children's Hospital of Philadelphia. This biorepository houses over 2,000 cases representing more than 30 types of pediatric brain tumors, aiming to accelerate understanding and personalized treatment approaches for these malignancies [103].

In a parallel effort, Claire Jean-Quartier and collaborators have demonstrated the inspiring potential of various bioinformatics tools such as Network Analyst, Navigator, OmicsNet, WebGestalt, and Cytoscape in developing and visualizing protein-protein interaction networks. These networks are instrumental in analyzing genes relevant to brain cancers, including GBM, general glioma, and low-grade astrocytoma [104]. This approach exemplifies how big data analytics can be leveraged to gain deeper insights into the molecular underpinnings of brain tumors, potentially informing more targeted and effective treatment strategies and sparking excitement about the future of brain tumor research.

The integration of AI in research and clinical applications has seen significant growth, with multitopic approaches playing an increasingly important role in achieving actual personalized PPM [105-108]. In the field of brain cancer, emerging omics, including radiomics, radiogenomics, and metabolomics, are showing promise. Radiomics has enhanced brain tumor diagnosis, staging, and therapy response prediction. Combined with genomics (radiogenomics), its utility is further expanded [105,106]. Textural features in radiomics have enabled researchers to differentiate between malignant and benign tissue [109], distinguish radiation necrosis from tumor recurrence [110], and discriminate between metastatic brain tumors and meningiomas [111].

**Figure 12:**
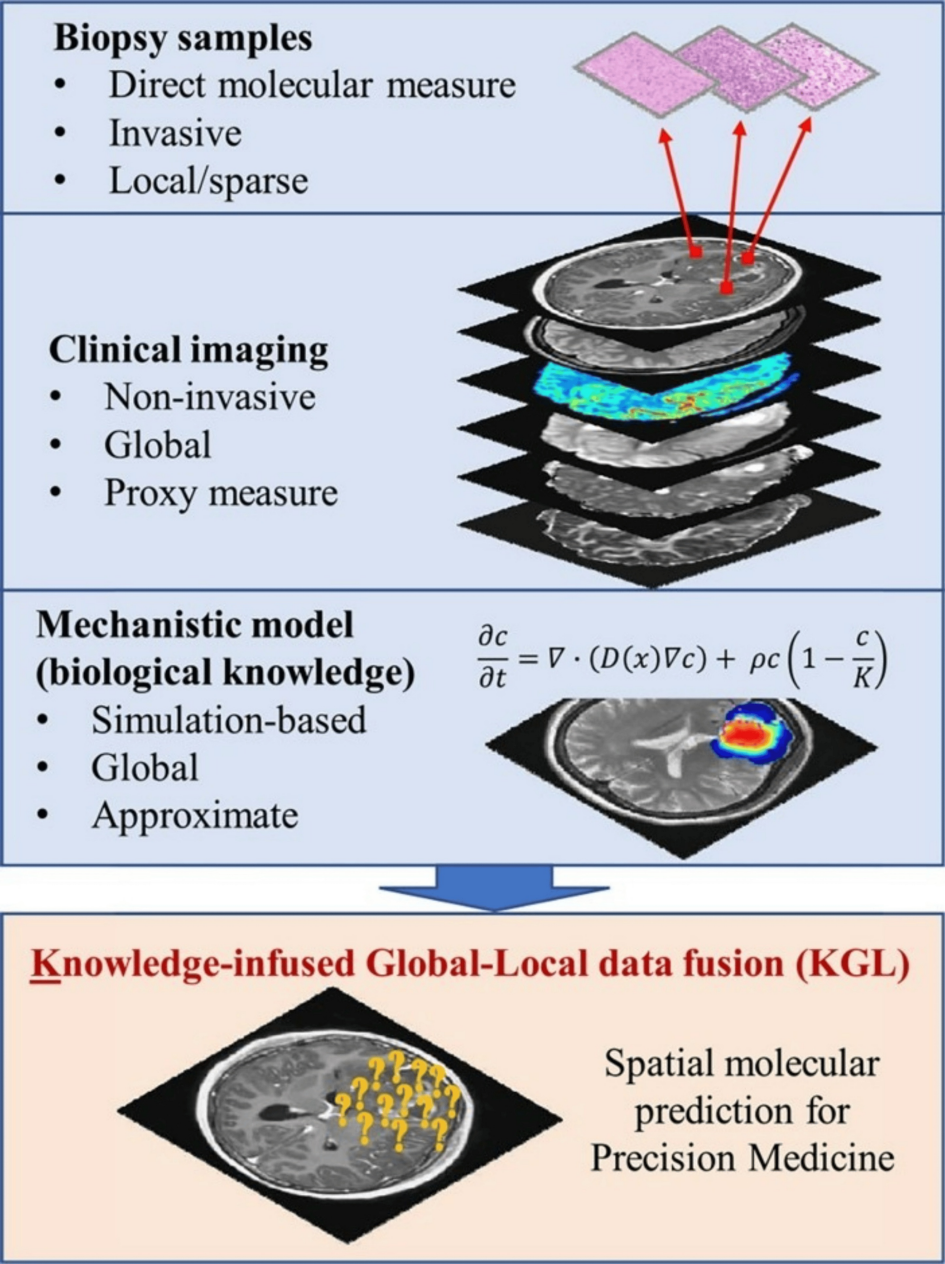
Illustration for combining biopsy, imaging, and clinical data to generate the knowledge-infused global-local method The formula in the image represents a mechanistic model based on biological knowledge. ∂c/∂t = ∇ · (D(x)∇c) + ρc(1 - c/K) : This is a partial differential equation that describes the change in concentration of cells or a chemical over time and space.The equation combines diffusion and logistic growth, which is common in biological systems. ∂c/∂t represents the rate of change of concentration (c) with respect to time (t); ∇ · (D(x)∇c) represents diffusion; ∇ is the del operator, which in this context represents spatial derivatives; D(x) is the diffusion coefficient, which may vary with position (x); ∇c represents the gradient of concentration; ρc(1 - c/K) represents the logistic growth; ρ (rho) is the growth rate; c is the concentration; K is the carrying capacity; (1 - c/K) ensures that growth slows as c approaches K. The image is reproduced from Wang et al. [108] and permission was obtained for the licensed content from the publisher, IEEE.

An emerging application of radiogenomics is using MRI imaging to detect the MGMT sequence associated with glioblastoma, contributing to PPM-guided prediction, diagnosis, and treatment strategies [105]. Meanwhile, metabolomics, which analyzes small molecules related to phenotypic expression, has spurred the development of new PPM-guided therapy approaches for brain cancer [107]. In a novel application of AI, Wang and colleagues [108] have developed a machine learning knowledge-infused global-local (KGL) model (Figure 12) to address the limitations of biopsy/MRI in providing appropriate spatial representation of brain tumors. This approach exemplifies how AI can overcome current diagnostic challenges and potentially improve treatment planning in neuro-oncology.

Brain tumor imaging has seen remarkable progress in recent years, driven by technological innovations and the integration of AI [109]. These advancements are revolutionizing how we detect, diagnose, and monitor brain tumors, paving the way for more precise and personalized treatment strategies. To address challenges such as the evolving brain tumor microenvironment, oncology within the framework of PPM necessitates advancements in imaging techniques. These include the use of MRI and positron emission tomography (PET) for identifying prognostic biomarkers in neuroinflammation contexts [110]. In evaluating brain tumor recurrence, 18F-FET PET/MRI may enhance the effectiveness of therapy [111]. Another PPM strategy for glioblastoma involves a metabolic imaging technique utilizing nicotinamide adenine dinucleotide phosphate (NADPH) fluorescence to predict therapy outcomes, as suggested by Morelli and colleagues [112].

Advances in therapeutic approaches: theranostics and nanotherapy

Theranostics tools offer a promising approach for enhancing localized drug delivery and monitoring treatment efficacy in gliomas, thus contributing to personalized therapy [113]. One such tool involves microbubbles linked to near-infrared fluorescence probes containing specific sequences, potentially improving targeted treatment and real-time monitoring.

Nanotherapeutic platforms, particularly biomimetic ones, present distinct advantages such as extended circulation time and enhanced targeting. These align with personalized PPM approaches for glioma therapy [114-116]. In the realm of imaging, albumin-based probes have shown improvements in diagnosis, phototherapy, and accuracy for image-guided procedures [115].

**Figure 13:**
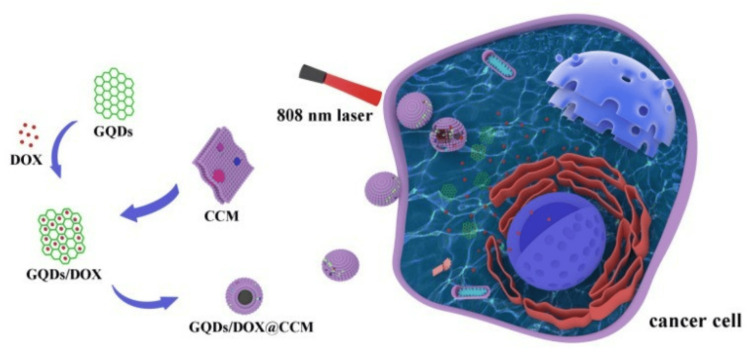
Depiction of biomimetic nanotherapeutic approach with quantum dots and chemotherapy. *Abbreviations: GQD: graphene quantum dot, DOX: doxorubicin, CCM: cancer cell membrane, nm: *nanometre The image is reproduced from Ren et al. [116] and available via Creative Commons Attribution 4.0 International License.

Ren and colleagues demonstrated a notable example of nanotherapeutics. They utilized graphene quantum dots combined with chemotherapy for glioma treatment. Their research, illustrated in Figure 13, revealed enhanced cancer cell uptake and improved tumor suppression efficacy [116]. These advancements in nanomedicine and theranostics represent significant strides toward more effective and personalized glioma treatments.

## Conclusions

The diagnosis and management of brain tumors present unique challenges in PPM. Recent advances like liquid biopsy, radiomics, and epigenetic studies have expanded our toolkit, offering non-invasive characterization, deeper insights into tumor biology, and new therapeutic targets. However, implementing these approaches faces hurdles such as resource constraints, complex tumor biology, and difficulties in conducting large-scale trials for rare subtypes. Despite these challenges, PPM in brain tumors holds profound potential. Integrating molecular profiling, advanced imaging, and targeted therapies promises improved outcomes and reduced unnecessary treatments. To fully realize PPM's potential, we must balance cutting-edge personalized approaches with population-level interventions, improving access to standard care and addressing disparities. Success depends on robust data-sharing, interdisciplinary collaboration, and continued research investment. While gene-environmental interactions in brain tumor etiology remain unclear, applying comprehensive PPM approaches could lead to breakthroughs. By embracing the complexity of brain tumor management and striving for a balanced approach, we can significantly improve outcomes for patients with these challenging neoplasms.
